# Survey of West Nile virus infection in wildlife species in the Orinoquia region of Colombia

**DOI:** 10.3389/fmicb.2025.1548538

**Published:** 2025-02-25

**Authors:** Nubia E. Matta, Felipe Andrés Gaitán-Albarracín, Gustavo Andrés Fuentes-Rodríguez, Óscar Andrés Rodríguez-Fandiño, Iván F. Calixto-Botía, Lady Johana Correa-Higuera

**Affiliations:** ^1^Laboratory Host-parasite relationship, Departamento de Biología, Facultad de Ciencias, Universidad Nacional de Colombia, Bogotá, Colombia; ^2^Laboratory of molecular studies of the Orinoquian region- LEMO, Facultad de Ciencias, Universidad Internacional del Trópico Americano, Universidad Internacional del Trópico Americano, Yopal, Colombia

**Keywords:** zoonotic, WNV, qPCR, NS5-3′NC, Casanare, savannas, vector-borne-disease

## Abstract

Studies focused on the epidemiological surveillance of arboviruses that cause potentially zoonotic diseases, such as dengue, Zika, or emerging viruses like West Nile virus (WNV), are critical due to their significant impact on public health. Although research on these infectious agents is increasing in Colombia, regions remain where the presence of zoonotic agents is still unknown. To address this knowledge gap, the present study aimed to investigate the current status of WNV circulation in wildlife in two municipalities of the department of Casanare (El Yopal and Paz de Ariporo) from the Colombian region of Orinoquia. Since the arrival of WNV in Colombia, reported in 2004, its detection has typically relied on antibody screening using ELISA. While informative, this technique needs to offer a sufficiently precise time frame to confirm active virus circulation. We employed a molecular approach to overcome this limitation, detecting WNV using qPCR, which provides greater specificity and a narrower time window. A total of 2,553 swab samples were collected from a broad sampling covering 142 birds, 19 mammals, and eight reptile species during 2023 and 2024 across four sampling events conducted during both the dry and wet seasons. The sampling included species with ecological or symbolic value to the region and those with economic importance, such as species used for human consumption (bushmeat). No evidence of WNV was detected in the evaluated species, indicating that these species were not infected with the virus during the sampling periods or that viral loads were below the detection threshold. Our results underscore the importance of further studies, including complementary diagnostic methods, such as antibody detection, to better understand the broader temporal infections and provide a more complete understanding of virus circulation.

## 1 Introduction

*Orthoflavivirus* comprises a group of arthropod-borne viruses that cause infections in mammals, birds, and reptiles (Postler et al., 2023). The most prominent *orthoflaviviruses* include Dengue, Zika, and Yellow fever, which collectively infected approximately 58 million people worldwide in 2021 (Liang and Dai, 2024). The primary vectors responsible for the transmission of these viruses are mosquitoes of the genus *Culex*, which are typically ornithophilic; mosquitoes of the genus *Aedes*, which primarily feed on mammals; and ticks of the genus *Ixodes*, which are generalists (Bakker et al., 2024; Ren et al., 2024). Within this genus, the West Nile virus (WNV) is notable for its zoonotic nature, with birds serving as its primary reservoirs. However, it has also been detected in other animals, including horses, sheep, reptiles, cats, rodents, American alligators, and marine crocodiles, all of which can act as amplifiers or reservoirs of the virus (Habarugira et al., 2020; Klenk et al., 2004).

This virus was first isolated in Uganda (Smithburn et al., 1940) and is a cosmopolitan arbovirus, present on all continents except Antarctica. According to Saiz (Saiz et al., 2021), the molecular classification of WNV lineages is based on phylogenetic relationships identified from complete WNV genomes and by geographic origin. To date, approximately nine lineages have been identified as follows Lineage one (L1), composed of three sublineages: L1a, present in Africa, Europe, and the Americas; L1b (Kunjin virus stain MRM61C- GenBank D00246), present in Australia and sharing 87% nucleotide identity and 97% amino acid (AA) identity with the L1 sublineages (Lanciotti et al., 2002); and L1c (putative lineage 5), present in India. Lineage 2 is found in Africa, Asia and Europe. L1 and L2 share only 76.8% nucleotide identity and 94.0% AA identity (Lanciotti et al., 2002), and are the most geographically widespread, virulent, and responsible for most outbreaks of infection (Thiiru et al., 2024). In contrast, lineages three through nine are considered to be country specific. For example, lineage 3 (Rabengsburg virus) was found in Culex pipiens in Southern Moravia (Czech Republic) (Bakonyi et al., 2005). In 2023, lineage 3 was identified in a human patient co-infected with lineage L1a in the United States (Davis et al., 2024). Lineage 4 is present in Russia; lineage 6 (GenBank GU047875) is found in Spain (Rizzoli et al., 2015; Vazquez et al., 2010); lineage 7 (Koutango virus) is found in Senegal (Charrel et al., 2003); lineage 8 has been identified in Senegal; and lineage 9 is found in Austria (Rizzoli et al., 2015, Saiz et al., 2021).

In South America, the circulation of WNV has been reported using two approaches. The most common approach is to detect antibodies (Abs) through ELISA analysis at the serological level. This method evaluates the presence or absence of IgG, indicating exposure to the virus in the past, and IgM, which usually indicates an active infection (Ayadi et al., 2019; Raulino et al., 2021). IgM is typically detected 1 week after exposure to the virus and can be present in humans for up to 3 months. To confirm WNV infection, a four-fold increase in antibody titers should occur between the acute and convalescent stages of infection (Shi and Wong, 2003). Conversely, the presence of WNV-specific IgG is typically identified shortly after IgM and persists for an extended period. Consequently, detecting IgG alone is a marker for prior infection, providing valuable insights into the prevalence and geographic distribution of WNV within populations (Shi and Wong, 2003). However, ELISA cannot discriminate the specific lineage of WNV (Kalaiyarasu et al., 2016; Mohammed et al., 2023). On the other hand, a molecular approach involves the detection of WNV by qPCR-RT, which can be detected on average up to 4 days before IgM (Costa et al., 2021; Martins et al., 2019; Morales et al., 2006; Osorio et al., 2012). This method is highly sensitive and specific, allowing for the direct detection of viral RNA in clinical or environmental samples, such as blood, cerebrospinal fluid, or mosquito pools. Furthermore, it enables active surveillance, particularly during outbreaks, as it facilitates real-time monitoring of viral activity and lineage distribution (Martins et al., 2019).

Between 2004 and 2025, Colombia reported several findings regarding the presence of WNV. In 2004, a study detected WNV antibodies in 9% of horses sampled in the Caribbean region, indicating the circulation of the virus among equine populations (Mattar et al., 2005). One hypothesis suggests that the spread of WNV throughout North and South America is likely due to the migration of birds. However, a study conducted on migratory and endemic birds in the Caribbean region of Colombia, a known route for boreal species migration, did not detect WNV in 300 birds examined (Soler-Tovar and Vera, 2011). Almost 10 years after the first report of WNV in Colombia, the virus was detected and sequenced in a population of flamingoes living in captivity at the zoo in the capital of the Antioquia department. This confirmed the presence of WNV in Colombia (Osorio et al., 2012). An important part of their findings was establishing a phylogenetic relationship between the strain present in Colombia and a strain previously reported in Louisiana, United States, possibly introduced through the migration of infected birds (Osorio et al., 2012). In 2015, WNV sequences were detected in studies conducted in the northern region of Colombia, in the department of Córdoba, which cluster with the attenuated Texas 2002 genotype in the species *Culex (Melanoconion) erraticus*, using RT-PCR (López et al., 2015). Finally, in 2023, the presence of WNV and other flaviviruses was reported in the municipality of Puerto Carreño, in the Eastern Plains of Colombia, and they detected WNV in the species *Culex browni* (Martínez et al., 2023).

Progress has been made over the past 20 years through studies focused on characterizing WNV in both wildlife and domestic animals using serological analysis (Góez-Rivillas et al., 2010; López et al., 2015; Mattar et al., 2005; Ruiz-Saenz et al., 2023; Soler-Tovar and Vera, 2011), with only a limited number addressing molecular detection through qPCR (Barajas et al., 2020; Góez-Rivillas et al., 2010; Mattar et al., 2011; Osorio et al., 2012; Soler-Tovar and Vera, 2011). These studies have provided evidence of lineage 1 of WNV circulation in some areas of Colombia. Although no human cases have been reported, this suggests a potential risk of zoonotic transmission (Barajas et al., 2020; Góez-Rivillas et al., 2010; Mattar et al., 2005; Osorio et al., 2012). Additionally, the presence of vectors such as mosquitoes from the *Culicidae* family circulating in the region (Miranda et al., 2019) makes Colombia ideal for developing and maintaining WNV transmission.

Colombia is also considered megadiverse (Instituto Humboldt, 2023) in terms of birds, mammals, amphibians, and reptiles, with migratory birds, which are potential agents of virus spread (Ain-Najwa et al., 2020). Specifically, the Orinoquia region of Colombia integrates three biogeographic systems: the piedmont, the alluvial plains, and the altiplano (Molina and Triana, 2011). Casanare, one of the departments in Colombia sharing the Orinoquia region, is characterized by temperatures ranging between 22°C and 27°C, with rainy periods between April and October (average rainfall 304.9 mm) and dry periods between December and March (average rainfall 51.9 mm) (FEDEARROZ, 2025). This department recorded 507 species of birds, 65 species of reptiles, 200 species of mammals, and 49 species of amphibians (Usma Oviedo and Trujillo González, 2011). This megadiversity of birds, the arrival of migratory birds, the average temperature, and rainfall patterns all appear to provide a scenario with high potential for establishing the WNV cycle or other pathogens with zoonotic potential. Wildlife hunting is also common in the region. Hunters are at increased risk of WNV infection primarily through mosquito bites and handling infected animal tissues rather than from eating the meat itself, as cooking eliminates the risk of transmission. Although consuming bushmeat hasn’t been proven to transmit WNV, hunters can be exposed to the virus through open wounds or contact with secretions from hunted animals (USGS, 2021). Hunters must wash their hands after handling carcasses and ensure that meat is thoroughly cooked to minimize risk (USGS, 2021).

This study aimed to assess the presence of WNV in wildlife from two localities in the Casanare department, located in the Orinoquia region of Colombia, using a qPCR approach. This research provides a valuable opportunity to investigate WNV circulation, including the highly pathogenic L1a lineage, which has been reported in America, within poorly understood yet highly biodiverse environments. For this purpose, the screening included birds, mammals, and reptiles of ecological value or that may be hunted by humans, with potential public health implications, given the risk of zoonotic spillover in communities reliant on wildlife for sustenance. This study contributes to understanding WNV epidemiology in tropical ecosystems and underscores the importance of integrating ecological and public health frameworks in biodiversity-rich, under-researched regions like the Orinoquia.

## 2 Materials and methods

### 2.1 Sample collection

A total of 1,216 cloacal swabs were obtained from bird species captured using mist nets, sweep nets, or direct nocturnal capture methods. Direct capture was conducted for reptiles using mist nets, trawls, and baited Tomahawk traps, yielding 812 cloacal swabs. In the case of mammals, large mammals were captured in pens or cages (Supplementary Table 1), while flying mammals were captured at night using mist nets, resulting in the collection of 525 rectal swabs. The swabs were preserved in DNA/RNA Shield^®^ and stored at −80°C until processing. Four field trips (Table 1) were conducted to sample bird species (cloacal swabs), mammals (rectal swabs), and herps (cloacal swabs). Sampling was carried out during the dry or rainy season between 2023 and 2024 in the municipalities of Paz de Ariporo and El Yopal in the department of Casanare, Colombia. Paz de Ariporo is defined by its extensive natural savannas and wetlands, which have significant biodiversity, including habitats for migratory and resident bird species despite human interventions associated with agriculture, extensive cattle ranching, and oil extraction. Sampling spots in El Yopal, on the other hand, due to proximity to a major population center, seem to have a higher degree of anthropic intervention (Usma Oviedo and Trujillo González, 2011).

**TABLE 1:** Total number of swab samples from birds, reptiles and mammals, that were processed by RT-PCR during each of the four field expeditions.

Taxonomic group	Sample type	Number of samples collected per expedition and season				Processed samples
		**March–April 2023 (Dry)**	**June 2023 (Rainy)**	**February–March 2024 (Dry)**	**June–July 2024 (Rainy)**	**Total**
Mammals	Rectal swabs	118	66	132	209	525
Reptiles	Cloacal swabs	206	197	211	198	812
Birds	Cloacal swabs	269	300	286	361	1216
Total		593	563	629	768	2553

### 2.2 DNA/RNA extraction and TaqMan qPCR multiplex

**FIGURE 1:**
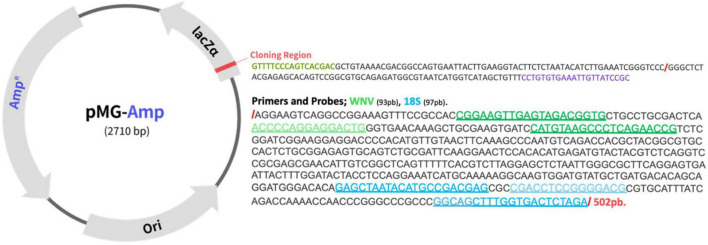
Sequence of the plasmid used as control for 18S and West Nile virus (WNV). The PCR target region with WNV (NS5-NC3′) and 18S sequences were designed to be cloned into a pMG-Amp cloning vector by Macrogen Inc. (Seoul, Korea). In green are the regions of annealing for WNV Primers and Probe and in Blue for 18S.

Total nucleic acids for molecular detection of 18S and WNV were extracted from the 2,553 swabs preserved in DNA/RNA Shield^®^ and maintained in the cold chain until processing. According to the manufacturer’s instructions, the kit used was the Quick-DNA/RNA™ MagBead (Cat. No. R2130; Zymo Research, CA, United States). WNV detection was carried out by TaqMan qPCR multiplex. The primers and probe for WNV were synthesized from the report (Vázquez et al., 2016) thus: forward 5′-CGGAAGTYGRGTAKACGGTGCTG-3′, reverse 5′-CGGTWYTGAGGGCTTACRTGG-3′, probe 5′-FAM- WCCCCAGGWGGACTG-BHQ1-3′. Our WNV detection primers target the 3′NC conserved fragment adjacent to NS5, which amplifies a 93 bp fragment in WNV lineage L1a (NC_009942.1, AF404756.1). These primers can also recognize the L1b (LC802098.1, D00246 Kunjin virus’), L2 (NC_001563.2, AY532665.1), L4 (AY277251.1) and L9 (KJ831223.1) lineages. It is important to note that these primers do not recognize the L1c (DQ256376.1 “Indian putative L5′’), L3 (AY765264.1 “Rabengsburg”) and L8 (KY703856.1 “Koutango virus”) lineages. As an internal control, we used primers to amplify a 97 bp fragment of 18S rRNA from the hosts (Zyrianova and Zaripov, 2022) as follows: forward 5′-GAGCTAATACATGCCGACGAG-3′, reverse 5′-CTAGAGTCACCAAAGCTGCC-3′, probe 5′-HEX-CGACCTCCGGGGACG-BHQ1-3′. A plasmid was also designed as a positive control for 18S and WNV; the PCR target region was designed with WNV and 18S sequences and cloned into a pMG-Amp cloning vector from Macrogen Inc. (Seoul, Korea) (Figure 1).

The Luna^®^ Universal Probe One-Step RT-qPCR Kit was used for the TaqMan qPCR multiplex (New England Biolabs-NEB), adding 9 μL of Luna reaction mix, 1 μL of Luna enzyme mix, 0,8 μM of WNV primers, 0,4 μM of WNV probe and 18S primers and probe, and 5 μL of RNA extraction, into 20 μL of final volume. Amplification conditions consisted of an initial step of retro transcription to 55°C for 10 min, denaturalization of 10 min at 95°C and 40 cycles of 15 s at 95°C, and 1 min at 60°C for annealing and extension. This step measures fluorescence to determine the cycle threshold (Ct) and evaluate the positivity. The different samples processed were pooled by species according to frequency, with a maximum of five samples per pool; if only one species was available, it was processed as a single sample without being grouped.

## 3 Results

A total of 2,553 cloacal (birds and reptiles) and rectal (mammals) swabs were collected in the municipalities of El Yopal and Paz de Ariporo, Casanare department, Colombia (Table 1). Birds were the most sampled group, associated with diurnal and nocturnal sampling efforts, followed by reptiles and mammals (Supplementary Table 1). In total, 142 species of birds, eight reptiles, and 19 mammals were collected (Supplementary Table 1). The sampling of reptiles and mammals focused mainly on species close to the local communities or species that are “object of hunting.”

For birds, sampling was expanded to include migratory species and other wild birds, as they are considered the primary reservoirs and amplifiers of the virus. Of particular importance due to the virus cycle and its association with boreal migratory birds, during this expedition we captured six boreal migratory species *Buteo albonotatus* (Order: Accipitriformes), *Calidris minutilla*, *Tringa solitaria* (Charadriiformes), *Parkesia noveboracensis*, *Catharus ustulatus* and *Tyrannus savana* (Passeriformes).

**FIGURE 2:**
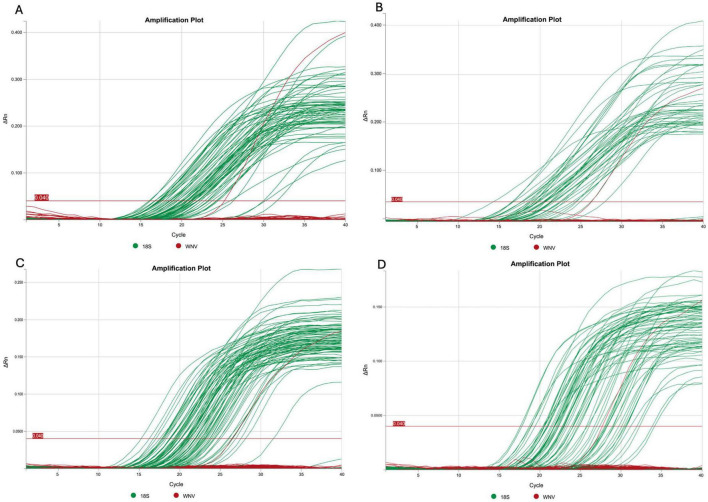
Representative qPCR amplification plots generated from pooled bird samples as follows: **(A)** some pools from the first expedition, **(B)** the second expedition, **(C)** the third expedition, and **(D)** the fourth expedition. For each plot, the Tm temperature was determined using QuantStudio Design and Analysis Software (D2). The 18S rRNA gene is shown in green and WNV is shown in red (with the red signal corresponding to the positive control).

All samples (*n* = 2,553) were negative for WNV detection using the qPCR approach. The 18S rRNA internal control was successfully amplified in all the samples tested (Figure 2).

## 4 Discussion

Zoonotic diseases are increasingly emerging as significant public health concerns due to environmental changes, human encroachment into natural habitats, and the complex interactions between wildlife, vectors, and humans. Due to the past detection of L1a WNV in Colombia, we analyzed the presence of this virus in birds, mammals, and reptiles in the Casanare Department in Colombia, a region of high biodiversity and ecological importance. No active WNV infections were detected in the swab samples.

The epidemiological survey carried out in this study, which was included 142 bird species, eight reptiles, and 19 mammals, found no evidence of current circulation of L1a WNV, nor of the L1b, L2, L4, and L9 lineages, in the wildlife of Casanare between 2023 and 2024. Given that in Colombia, there are previous reports of the virus in wildlife and possible vectors (Martínez et al., 2023; Mattar et al., 2005; Osorio et al., 2012).

The negative results are surprising due to the inclusion of a wide variety of species and the large number of animals sampled, including possible reservoir species such as wild birds, waterfowl or migratory birds. However, this negative result contrasts with those obtained in the United States, where various outbreaks have already been detected, even human cases. A possible explanation for this result may be the so-called dilution effect (Civitello et al., 2015). Since this region is megadiverse in fauna, the chances of encounter between the virus, the insect vector and susceptible hosts decrease. This effect, for example, explains the lower prevalences of blood parasites in the Neotropics compared to the Nearctic (LaPointe et al., 2012). Another possible explanation for this phenomenon involves a protection effect from previous exposure to other flaviviruses or even the circulation of attenuated strains of WNV (López et al., 2015). Despite this, it is essential to highlight that even though our qPCR results did not detect WNV in the fauna studied, it does not mean that WNV is not circulating now or in the past in this region. This is partly because qPCR only detects active infections, and the duration of viremia is short, limiting the identification of possible infected hosts (Hofmeister et al., 2018; Trogu et al., 2021).

On the other hand, the 18S rRNA internal control was successfully amplified in all samples analyzed, indicating the integrity of the genetic material analyzed (Figure 2). This robust amplification allows us to be confident in the results obtained by qPCR. Additionally, given that the primer setup allows for the detection of certain specific lineages, it is important to consider that other lineages (L1c, L3, L6, L7, and L8), which are less relevant to health systems due to their reduced pathogenicity compared to L1a, L1b, and L2, may also be present. However, their likelihood is presumed to be low, as they have not yet been reported in the Americas (Costa et al., 2021; Martins et al., 2019).

Among birds, the primary vertebrate hosts that amplify WNV, certain orders are frequently reported as virus carriers. Our sampling covered these orders, with a substantial number of samples, allowing us to conduct a thorough screening across a diverse range of bird populations, including Passeriformes (n: 390), Charadriiformes (n:175), Anseriformes (n:162), Galliformes (n:99), Columbiformes (n: 76), Pelecaniformes (n:22), Accipitriformes (n:12), Strigiformes (n:21), Falconiformes (n:1) (Supplementary Table 1). Notably, in Colombia, there is a report of a WNV L1a found in Pelecaniformes in *Phoenicopterus ruber*, isolated in the Medellín Zoo (Osorio et al., 2012). Our results coincide with those Soler-Tovar and Vera (Soler-Tovar and Vera, 2011) reported, analyzing resident and migratory birds in the San Andres islands (Colombia), where no active and natural WNV infection was reported.

In comparison with other geographical areas characterized by frequent WNV outbreaks, Trogu et al. (2021) analyzed Passeriformes, Columbiformes, Charadriiformes, and Accipitriformes and found prevalences ranging from 3.1% to 16% in Italy. Meanwhile, serological survey in Charadriiformes in the same country reported a frequency of 57% (4/7) (Di Girolamo et al., 2016). Passeriformes were also screened in Germany by molecular tools with variable frequencies of 1,8%–6,5% (Andersen et al., 2024; Pauvolid-Corrêa et al., 2014). These results imply that other biotic and abiotic factors may be favoring the establishment and permanence of this infectious agent by cycles of infection–reinfection in wild bird populations in certain regions of the planet for this group of vertebrates.

Continuing with reptiles, a significant number of iguanas (Table 1) were sampled in the present study. The *iguana iguana* has been used as an experimental model for WNV infection, and the authors’ findings suggest that it is a potential reservoir of the virus (Klenk and Komar, 2003). Regarding testudines, this is the first study in Colombia that evaluates the presence of this virus in a broad survey involving 441 wild turtles (*Podocnemis vogli, unifilis, Chelus orinocensis*) and 74 tortoises (*Chelonoidis carbonaria*), which is comparable to recent studies done in Italy where they did not report the presence of this pathogen in any of the turtles analyzed, either by qPCR (0/41) or by serological techniques (0/33) (Di Girolamo et al., 2016). On the other hand, studies have been reported from the Nearctic on organisms of the order Crocodylia, showing that they act as reservoirs for infection (Andersen et al., 2024). However, for the Neotropics, the most recent reports do not detect the presence of antibodies to this virus in local populations of *Caiman crocodilus* in Brazil, where no positive serological results were obtained (Pauvolid-Corrêa et al., 2014).

Casanare is characterized by hosting one of the highest concentrations of mammal populations in the country (representing 15% of the country’s diversity), with areas such as wetlands that can host thousands of capybaras (*Hydrochaeris hydrochaeris*) (Usma Oviedo and Trujillo González, 2011). This rodent is highly sought after for its skin and meat in Colombia, Venezuela, Argentina, and Uruguay, and it has been reported positive for the orthoflavivirus Cacipacoré in Brazil (Figueiredo et al., 2017). Likewise, equids tested positive for WNV in Colombia (Mattar et al., 2005). It is important to note that mammals are considered to be a dead end in the WNV transmission cycle (Ahlers and Goodman, 2018). However, different orders have been identified as susceptible to WNV infection, such as Pilosa, Chiroptera, Primates, and Rodentia (García-Romero et al., 2023). Chaves et al. (2021) reported the presence of WNV in Costa Rica at 2.3 % (2/86) in non-human primates. Additionally, WNV infections in the order Chiroptera have been widely reported in the Old and New World, with WNV exposure evident in species such as *Eptesicus fuscus* or *Myotis lucifugus* in North America or *Rousettus aegyptiacus* in Africa and Asia (Root, 2013). Although in Colombia, WNV has not been detected in bats, they are key species to study, given that species such as *Carollia perspicillata* or *Phyllostomus discolor* have been identified as reservoirs of arboviruses such as dengue (Calderón et al., 2019).

Colombia reports 324 species of *Culex* mosquitoes, which suggests a high likelihood of establishing a WNV transmission cycle in the country (Martínez et al., 2023). Casanare experiences distinct rainy and dry seasons, with temperatures ranging between 22°C and 27°C, factors that influence the population dynamics of *Culex*, the primary vector of WNV. Recent studies in the Orinoquia region using culicine virome metagenomics have identified WNV in *Culex browni* in Puerto Carreño-Vichada (Martínez et al., 2023). It would be crucial to gather more information about the circulating vectors in the area and their seasonal abundance to better understand the transmission dynamics of WNV and other vector-borne diseases with zoonotic potential. Most epidemiological surveillance studies of viruses in wildlife first assess the presence of antibodies by serological tests. This could identify past infections with IgG or recent infections with IgM. Based on such reports, sampling could focus on those species with antibody responses (Lorenz and Chiaravalloti-Neto, 2022).

Zoonotic diseases are becoming more prevalent due to the increase in negative human interventions in natural ecosystems, climate change, and their close relationship with domestic and wild fauna at the rural level. Within the group of zoonotic diseases in Colombia, arboviruses are on the rise, including historically reported viruses such as Dengue, Zika, or Chikungunya, as well as recently reported or re-emerging viruses in the national territory, such as Pichindeì virus (Arenavirus), Mayaro virus (Alphavirus) and Oropouche virus (Bunyavirus). Due to their rapid spread, these viruses are considered potential candidates as aetiological agents of future epidemics in the Americas (Martínez et al., 2023).

Although our approach allowed us to detect different lineages in addition to L1a, the virus was not found to be actively circulating in the samples analyzed. Given the environmental conditions and the accelerated climate change, we suggest that entities such as the Ministry of Health, the Ministry of Environment, health secretariats, health centers, schools and universities actively provide education, socialization and awareness to the local population to prevent and warn of the zoonotic risks associated with the increasingly frequent contact between the local population and wildlife, or risks associated with activities such as hunting, which is an ancestral practice in the region. On the other hand, surveillance in Colombia will be maintained by increasing the number of serological studies and entomovirological surveys.

## Data Availability

The original contributions presented in this study are included in this article/Supplementary material, further inquiries can be directed to the corresponding author.

## References

[B1] Ahlers L. R. Goodman A. (2018). The immune responses of the animal hosts of west nile virus: A comparison of insects, birds, and mammals. *Front. Cell. Infect. Microbiol.* 8:96. 10.3389/fcimb.2018.00096 29666784 PMC5891621

[B2] Ain-Najwa M. Yasmin A. Omar A. Arshad S. Abu J. Mohammed H. (2020). Evidence of west nile virus infection in migratory and resident wild birds in west coast of Peninsular Malaysia. *One Health* 10:100134. 10.1016/j.onehlt.2020.100134 32405525 PMC7210594

[B3] Andersen D. Ann Fischer G. Combrink L. (2024). The alligator and the mosquito: North American crocodilians as amplifiers of west nile virus in changing climates. *Microorganisms* 12:1898. 10.3390/microorganisms12091898 39338572 PMC11433929

[B4] Ayadi T. Hammouda A. Beck C. Boulinier T. Lecollinet S. Selmi S. (2019). Flaviviruses in migratory passerines during spring stopover in a desert oasis. *Zoonoses Public Health* 66 495–503. 10.1111/zph.12584 31090178

[B5] Bakker J. Münger E. Esser H. Sikkema R. de Boer W. Sprong H. (2024). Ixodes ricinus as potential vector for usutu virus. *PLoS Neglected Trop. Dis.* 18:e0012172. 10.1371/journal.pntd.0012172 PMC11236205 38985837

[B6] Bakonyi T. Hubálek Z. Rudolf I. Nowotny N. (2005). Novel flavivirus or new lineage of west nile virus. *Central Europe. Emerg. Infect. Dis.* 11 225–231. 15752439 10.3201/eid1102.041028 PMC3320449

[B7] Barajas P. Ciouderis A. Garcia D. Gongora A. (2020). Vigilancia epidemiológica Al virus del oeste del nilo en municipios del departamento del meta. *Revista MVZ Córdoba* 25:1252.

[B8] Calderón A. Guzmán C. Mattar S. Rodriguez V. Martínez C. Violet L. (2019). Dengue virus in bats from córdoba and sucre. *Colombia. Vector Borne Zoonotic Dis.* 19 747–751. 31211661 10.1089/vbz.2018.2324 PMC6765209

[B9] Charrel R. Brault A. Gallian P. Lemasson J. Murgue B. Murri S. (2003). Evolutionary relationship between old world west nile virus strains. evidence for viral gene flow between Africa, the Middle East, and Europe. *Virology* 315 381–388. 10.1016/s0042-6822(03)00536-1 14585341

[B10] Chaves A. Piche-Ovares M. Ibarra-Cerdeña C. Corrales-Aguilar E. Suzán G. Moreira-Soto A. (2021). Serosurvey of nonhuman primates in costa rica at the human–wildlife interface reveals high exposure to flaviviruses. *Insects* 12:554. 10.3390/insects12060554 34203687 PMC8232092

[B11] Civitello D. Cohen J. Fatima H. Halstead N. Liriano J. McMahon T. (2015). Biodiversity inhibits parasites: Broad evidence for the dilution effect. *Proc. Natl. Acad. Sci.* 112 8667–8671. 10.1073/pnas.1506279112 26069208 PMC4507196

[B12] Costa ÉA. Giovanetti M. Catenacci L. Fonseca V. Aburjaile F. Chalhoub F. (2021). West nile virus in Brazil. *Pathogens* 10:896. 34358046 10.3390/pathogens10070896 PMC8308589

[B13] Davis E. Velez J. Hamik J. Fitzpatrick K. Haley J. Eschliman J. (2024). Evidence of lineage 1 and 3 west nile virus in person with neuroinvasive disease. Nebraska, USA, 2023. *Emerg. Infect. Dis.* 30 2090–2098. 10.3201/eid3010.240595 39320165 PMC11431902

[B14] Di Girolamo N. Selleri P. Di Gennaro A. Maldera M. Nardini G. Morandi B. (2016). Lack of detection of West Nile Virus in an islander population of chelonians during a West Nile virus outbreak. *Vet. Italiana* 52 159–173. 10.12834/VetIt.356.1600.2 27393879

[B15] FEDEARROZ, (2025). Condiciones Climatológicas Históricas de La Precipitación Acumulada. Servicio Climatico Para el Cultivo Del Arroz. Available online at: https://clima.fedearroz.com.co/historico-region/ (accessed December 18, 2024).

[B16] Figueiredo M. Amarilla A. Figueiredo G. Alfonso H. Lippi V. Maia F. (2017). Cacipacore virus as an emergent mosquito-borne flavivirus. *Rev. Soc. Bras. Med. Trop.* 50 539–542. 10.1590/0037-8682-0485-2016 28954077

[B17] García-Romero C. Carrillo Bilbao G. Navarro J. Martin-Solano S. Saegerman C. (2023). Arboviruses in mammals in the neotropics: A systematic review to strengthen epidemiological monitoring strategies and conservation medicine. *Viruses* 15:417. 10.3390/v15020417 36851630 PMC9962704

[B18] Góez-Rivillas Y. Taborda N. Javier Diaz F. Gongora A. Rodas D. Sáenz D. (2010). Antibodies to West nile virus in equines of antioquia and meta, Colombia, 2005-2008. *Rev. Colomb. Ciencias Pecuarias* 23 462–470.

[B19] Habarugira G. Moran J. Colmant A. Davis S. O’Brien C. Hall-Mendelin S. (2020). Mosquito independent transmission of west nile virus in farmed saltwater crocodiles (Crocodylus Porosus). *Viruses* 12:198. 10.3390/v12020198 32054016 PMC7077242

[B20] Hofmeister E. K. Lund M. Bochsler V. (2018). West nile virus infection in american singer canaries: An experimental model in a highly susceptible avian species. *Veterinary Pathol.* 55 531–538. 10.1177/0300985818760377 29506438

[B21] Instituto Humboldt, (2023). *Reporte de Estado y Tendencias de La Biodiversidad Continental de Colombia 2023.* Available online at: https://reporte.humboldt.org.co/biodiversidad/2023/ (accessed December 8, 2024).

[B22] Kalaiyarasu S. Mishra N. Khetan R. Singh V. (2016). Serological evidence of widespread west nile virus and Japanese encephalitis virus infection in native domestic ducks (*Anas Platyrhynchos* Var Domesticus) in kuttanad region, Kerala, India. *Comp. Immunol. Microbiol. Infect. Dis.* 48 61–68. 10.1016/j.cimid.2016.08.002 27638121

[B23] Klenk K. Komar N. (2003). Poor replication of west nile virus (New York 1999 Strain) in three reptilian and one amphibian species. *Am. J. Trop. Med. Hyg.* 69 260–262. 14628941

[B24] Klenk K. Snow J. Morgan K. Bowen R. Stephens M. Foster F. (2004). Alligators as West Nile virus amplifiers. *Emerg. Infect. Dis.* 10 2150–2155. 10.3201/eid1012.040264 15663852 PMC3323409

[B25] Lanciotti R. Ebel G. Deubel V. Kerst A. Murri S. Meyer R. (2002). Complete genome sequences and phylogenetic analysis of West Nile virus strains isolated from the United States, Europe, and the Middle East. *Virology* 298 96–105. 10.1006/viro.2002.1449 12093177

[B26] LaPointe D. Atkinson C. Samuel M. (2012). Ecology and conservation biology of avian Malaria. *Ann. N. Y. Acad. Sci.* 1249 211–226. 22320256 10.1111/j.1749-6632.2011.06431.x

[B27] Liang Y. Dai X. (2024). The global incidence and trends of three common flavivirus infections (dengue, yellow fever, and zika) from 2011 to 2021. *Front. Microbiol.* 15:1458166. 10.3389/fmicb.2024.1458166/full 39206366 PMC11349664

[B28] López R. Soto S. Gallego-Gómez J. (2015). Evolutionary relationships of west nile virus detected in mosquitoes from a migratory bird zone of Colombian Caribbean. *Virol. J.* 12:80. 10.1186/s12985-015-0310-8 25989901 PMC4445300

[B29] Lorenz C. Chiaravalloti-Neto F. (2022). Why Are there no human west nile virus outbreaks in South America?”. *Lancet Regional Health Am.* 12:100276. 10.1016/j.lana.2022.100276 PMC9903813 36776433

[B30] Martínez L. Silva E. Casseb L. Silva S. Cruz A. Pantoja J. (2023). Employing oxford nanopore technologies (ONT) for understanding the ecology and transmission dynamics of flaviviruses in mosquitoes (Diptera: Culicidae) from Eastern Colombia. *Acta Tropica* 245:106972. 10.1016/j.actatropica.2023.106972 37331645

[B31] Martins L. Silva E. Casseb L. Silva S. Cruz A. Pantoja J. (2019). First isolation of West Nile virus in Brazil. *Mem. Instituto Oswaldo Cruz* 114:e180332. 10.1590/0074-02760180332 PMC6343470 30672980

[B32] Mattar S. Edwards E. Laguado J. González M. Alvarez J. Komar N. (2005). West Nile Virus antibodies in Colombian horses. *Emerg. Infect. Dis.* 11 1497–1498. 10.3201/eid1109.050426 16673523 PMC3310636

[B33] Mattar S. Komar N. Young G. Alvarez J. Gonzalez M. (2011). Seroconversion for West Nile and St. Louis encephalitis viruses among sentinel horses in Colombia. *Mem. Instituto Oswaldo Cruz.* 106 976–979. 10.1590/s0074-02762011000800012 22241119

[B34] Miranda J. Mattar S. Gonzalez M. Hoyos-López R. Aleman A. Aponte J. (2019). First report of culex Flavivirus infection from culex coronator (Diptera: Culicidae), Colombia. *Virol. J.* 16:1. 10.1186/s12985-018-1108-2 30606229 PMC6318882

[B35] Mohammed M. Yasmin A. Ramanoon S. Noraniza M. Ooi P. Ain-Najwa M. (2023). Serological and molecular surveillance of West Nile Virus in domesticated mammals of Peninsular Malaysia. *Front. Vet. Sci.* 10:1126199. 10.3389/fvets.2023.1126199 37456951 PMC10343450

[B36] Molina N. Triana H. (2011). Panorama de La investigación en producción animal Desde La perspectiva Del entorno socioeconómico y cultural En La Región de Los {Llanos} {Orientales} y El {Casanare}. *Gestión Soc.* 4 31–44.

[B37] Morales M. Barrandeguy M. Fabbri C. Garcia J. Vissani A. Trono K. (2006). West Nile virus isolation from equines in Argentina, 2006. *Emerg. Infect. Dis.* 12 1559–1561. 17176571 10.3201/eid1210.060852 PMC3290965

[B38] Osorio J. Ciuoderis K. Lopera J. Piedrahita L. Murphy D. Levasseur J. (2012). Characterization of West Nile viruses isolated from captive American flamingoes (*Phoenicopterus Ruber*) in medellin, Colombia. *Am. J. Trop. Med. Hyg.* 87 565–572. 10.4269/ajtmh.2012.11-0655 22802436 PMC3435365

[B39] Pauvolid-Corrêa A. Campos Z. Juliano R. Velez J. Nogueira R. Komar N. (2014). Serological evidence of widespread circulation of West Nile Virus and other flaviviruses in equines of the Pantanal, Brazil. *PLoS Negl. Trop. Dis.* 8:e2706. 10.1371/journal.pntd.0002706 24551266 PMC3923745

[B40] Postler T. Beer M. Blitvich B. Bukh J. de Lamballerie X. Drexler J. (2023). Renaming of the genus flavivirus to Orthoflavivirus and extension of binomial species names within the family Flaviviridae. *Arch. Virol.* 168:224. 10.1007/s00705-023-05835-1 37561168

[B41] Raulino R. Thaurignac G. Butel C. Villabona-Arenas C. Foe T. Loul S. (2021). Multiplex detection of antibodies to Chikungunya, O’nyong-Nyong, Zika, Dengue, West Nile and usutu viruses in diverse nonhuman primate species from cameroon and the democratic republic of Congo. *PLoS Neglected Trop. Dis.* 15:e0009028. 10.1371/journal.pntd.0009028 33476338 PMC7853492

[B42] Ren N. Jin Q. Wang F. Huang D. Yang C. Zaman W. (2024). Evaluation of vector susceptibility in aedes aegypti and culex pipiens pallens to tibet orbivirus. *mSphere* 9:e0006224. 10.1128/msphere.00062-24 38530016 PMC11036799

[B43] Rizzoli A. Jimenez-Clavero M. Barzon L. Cordioli P. Figuerola J. Koraka P. (2015). The challenge of West Nile virus in Europe: Knowledge gaps and research priorities. *Euro Surveill. Eur. Communicable Dis. Bull.* 20:21135. 10.2807/1560-7917.es2015.20.20.21135 26027485

[B44] Root J. J. (2013). West Nile virus associations in wild mammals: A synthesis. *Arch. Virol.* 158 735–752. 23212739 10.1007/s00705-012-1516-3

[B45] Ruiz-Saenz J. Martinez-Gutierrez M. Pujol F. (2023). Multiple introductions of highly pathogenic avian influenza H5N1 Clade 2.3.4.4b into South America. *Travel Med. Infect. Dis.* 53:102591. 37201592 10.1016/j.tmaid.2023.102591

[B46] Saiz J. Martín-Acebes M. Blázquez A. Escribano-Romero E. Poderoso T. Jiménez (2021). Pathogenicity and virulence of West Nile virus revisited eight decades after its first isolation. *Virulence* 12 1145–1173. 10.1080/21505594.2021.1908740 33843445 PMC8043182

[B47] Shi P. Wong S. (2003). Serologic diagnosis of West Nile virus infection. *Exp. Rev. Mol. Diagnostics* 3 733–741. 10.1586/14737159.3.6.733 14628901

[B48] Smithburn K. Hughes T. Burke A. Paul J. (1940). *A Neurotropic Virus Isolated from the Blood of a Native of Uganda.* Availaable at: https://www.ajtmh.org/view/journals/tpmd/s1-20/4/article-p471.xml (August 3, 2024).

[B49] Soler-Tovar D. Vera V. (2011). Evaluación del virus del oeste del nilo en aves silvestres de una isla del caribe colombiano: Evaluation of west nile virus in wild birds on an Island in the Colombian Caribbean. *Ornitol. Colombiana* 14–20.

[B50] Thiiru J. Langat S. Mulwa F. Cinkovich S. Koka H. Yalwala S. (2024). Characterization of West Nile virus koutango lineage from phlebotomine sandflies in Kenya. *PLoS One* 19:e0301956. 10.1371/journal.pone.0301956 39173002 PMC11341046

[B51] Trogu T. Canziani S. Salvato S. Tolini C. Grilli G. Chiari M. (2021). Survey on the presence of viruses of economic and zoonotic importance in Avifauna in Northern Italy. *Microorganisms* 9:1957. 10.3390/microorganisms9091957 34576852 PMC8471648

[B52] USGS (2021). *Can Hunters Get West Nile Virus from Eating Infected Game Birds?.* Available online at: https://www.usgs.gov/faqs/can-hunters-get-west-nile-virus-eating-infected-game-birds (January 25, 2025).

[B53] Usma Oviedo J. Trujillo González F. (2011). *Biodiversidad Del Departamento de Casanare: Identificación de Ecosistemas Estratégicos.* Available online at: https://repositorio.unal.edu.co/handle/unal/9679 (accessed August 9, 2024).

[B54] Vázquez A. Herrero L. Negredo A. Hernández L. Sánchez-Seco M. Tenorio A. (2016). Real time PCR assay for detection of all known lineages of West Nile virus. *J. Virol. Methods* 236 266–270. 27481597 10.1016/j.jviromet.2016.07.026

[B55] Vazquez A. Sanchez-Seco M. Ruiz S. Molero F. Hernandez L. Moreno J. (2010). Putative new lineage of West Nile Virus, Spain. *Emerg. Infect. Dis.* 16 549–552. 20202444 10.3201/eid1603.091033 PMC3322021

[B56] Zyrianova I. Zaripov O. (2022). 18S ribosomal DNA-based PCR test for avian and mammalian DNA identification in meat products. *Vet. Anim. Sci*. 15:100234. 10.1016/j.vas.2022.100234 35112013 PMC8790660

