# Supplementary material for: Survey of West Nile virus infection in wildlife species in the Orinoquia region of Colombia
Source: Front Microbiol. 2025 Feb 25;16:1548538. doi: 10.3389/fmicb.2025.1548538 (PMC11893598; doi:10.3389/fmicb.2025.1548538)
Supplement: Supplementary file 1 [file Table_1.docx]

| **Group** | **Order** | **Family** | **Species** | **Common name** | | **Location** | | | **Number total of samples (swabs)** |
| --- | --- | --- | --- | --- | --- | --- | --- | --- | --- |
|  |  |  |  |  |  | **Yopal** | **Paz de Apiroro** | |  |
| Mammals | Artiodactyla | Bovidae | *Bos taurus* | Cow | | 37 | 19 | | 56 |
|  |  |  | *Bubalus bubalis* | Water Buffalo | | - | 2 | | 2 |
|  |  |  | *Capra hircus* | Domestic goat | | 9 | 16 | | 25 |
|  |  | Suidae | *Sus crofa domestica* | Pig | | 11 | 56 | | 67 |
|  | Carnivora | Canidae | *Cerdocyon thous* | Crab-eating Fox | | - | 2 | | 2 |
|  | Cingulata | Dasypodidae | *Dasypus sabanicola* | Llanos Long-nosed Armadillo | | 1 | 34 | | 35 |
|  | Didelphimorphia | Didelphidae | *Didelphis marsupialis* | Common opossum | | 24 | 14 | | 38 |
|  |  |  | *Marmosa robinsoni* | Robinson's mouse opossum | | - | 1 | | 1 |
|  |  |  | *Caluromys sp.* | Opossums | | - | 1 | | 1 |
|  | Lagomorpha | Leporidae | *Oryctolagus cuniculus* | Rabbit | | 2 | - | | 2 |
|  | Perissodactyla | Equidae | *Equus ferus caballus* | Horse | | 43 | 19 | | 62 |
|  | Pilosa | Myrmecophagidae | *Tamandua tetradactyla* | Southern Tamandua | | 4 | - | | 4 |
|  |  |  | *Myrmecophaga tridactyla* | Giant Anteater | | 14 | 6 | | 20 |
|  | Primates | Atelidae | *Alouatta seniculus* | Red howler monkey | | 1 | - | | 1 |
|  | Rodentia | Caviidae | *Hydrochoerus hydrochaeris* | Capybara | | 8 | 176 | | 184 |
|  |  | Cuniculidae | *Cuniculus paca* | Lowland Paca | | 3 | - | | 3 |
|  |  | Dasyproctidae | *Dasyprocta fuliginosa* | Black Agouti | | 2 | 16 | | 18 |
|  |  | Echimyidae | *Proechimys oconnelli* | O'Connell's Spiny Rat | | 2 | 1 | | 3 |
|  |  |  | *Proechimys semispinosus* | Tome's Spiny Rat | | 1 | - | | 1 |
| **Total group** | | | | |  | **162** | **363** | | **525** |
| Reptiles | Crocodylia | Alligatoridae | *Caiman crocodilus* | Common Caiman | | 92 | 111 | | 203 |
|  | Squamata | Boidae | *Eunectes murinus* | Green Anaconda | | 2 | - | | 2 |
|  |  | Iguanidae | *Iguana iguana* | Green Iguana | | 52 | 17 | | 69 |
|  |  | Teiidae | *Tupinambis teguixin* | Gold Tegu | | 7 | 16 | | 23 |
|  | Testudines | Chelidae | *Chelus orinocensis* | Orinoco Mata Mata | | - | 2 | | 2 |
|  |  | Testudinidae | *Chelonoidis carbonaria* | Red-footed Tortoise | | 51 | 23 | | 74 |
|  |  | Podocnemididae | *Podocnemis unifilis* | Yellow-headed Sideneck | | - | 4 | | 4 |
|  |  |  | *Podocnemis vogli* | Savanna Side-necked Turtle | | 117 | 318 | | 435 |
| **Total group** | | | | |  | **321** | | **491** | **812** |
| Birds | Accipitriformes | Accipitridae | *Buteo albonotatus cf* | Zone-tailed Hawk | | - | 1 | | 1 |
|  |  |  | *Rupornis magnirostris* | Roadside Hawk | | 1 | - | | 1 |
|  |  | Cathartidae | *Cathartes aura* | Turkey Vulture | | - | 2 | | 2 |
|  |  |  | *Coragyps atratus* | Black Vulture | | 2 | 5 | | 7 |
|  |  |  | *Sarcoramphus papa* | King Vulture | | 1 | - | | 1 |
|  | Anseriformes | Anatidae | *Amazonetta brasiliensis* | Brazilian Teal | | 2 | 10 | | 12 |
|  |  |  | *Anas platyrhynchos domesticus* | Domestic Mallard | | 3 | - | | 3 |
|  |  |  | *Anser anser* | Greylag Goose | | 2 | 1 | | 3 |
|  |  |  | *Cairina moschata* | Muscovy Duck | | 11 | 19 | | 30 |
|  |  |  | *Dendrocygna autumnalis* | Black-bellied Whistling Duck | | 5 | 43 | | 48 |
|  |  |  | *Oressochen jubatus* | Andean Goose | | 3 | 32 | | 35 |
|  |  |  | *Dendrocygna viduata* | White-faced Whistling-Duck | | 1 | - | | 1 |
|  |  |  | *Spatula discors* | Blue-winged Teal | | - | 31 | | 31 |
|  | Caprimulgiformes | Caprimulgidae | *Hydropsalis cayennensis* | White-tailed Nightjar | | 13 | 5 | | 18 |
|  |  |  | *Hydropsalis maculicaudus* | Spot-tailed Nightjar | | 2 | 1 | | 3 |
|  |  |  | *Chordeiles nacunda* | Nacunda Nighthawk | | 2 | 2 | | 4 |
|  |  |  | *Nyctidromus albicollis* | Common Pauraque | | 46 | 19 | | 65 |
|  |  |  | *Nyctiprogne leucopyga* | Band-tailed Nighthawk | | 1 | 1 | | 2 |
|  | Charadriiformes | Burhinidae | *Burhinus bistriatus* | Double-striped Thick-knee | | - | 1 | | 1 |
|  |  | Charadriidae | *Charadrius collaris* | Collared Plover | | - | 1 | | 1 |
|  |  |  | *Vanellus cayanus* | Pied Lapwing | | - | 19 | | 19 |
|  |  |  | *Vanellus chilensis* | Southern Lapwing | | 12 | 14 | | 26 |
|  |  | Jacanidae | *Jacana jacana* | Wattled Jacana | | 77 | 22 | | 99 |
|  |  | Recurvirostridae | *Himantopus mexicanus* | Black-necked Stilt | | 2 | 19 | | 21 |
|  |  | Scolopacidae | *Calidris minutilla* | Least Sandpiper | | - | 3 | | 3 |
|  |  |  | *Tringa solitaria* | Solitary Sandpiper | | - | 4 | | 4 |
|  | Ciconiiformes | Ciconiidae | *Jabiru mycteria* | Jabiru | | - | 1 | | 1 |
|  |  |  | *Mycteria americana* | Wood Stork | | - | 3 | | 3 |
|  | Columbiformes | Columbidae | *Columbina minuta* | Plain-breasted Ground Dove | | 10 | 5 | | 15 |
|  |  |  | *Columbina squammata* | Scaled Dove | | 7 | 8 | | 15 |
|  |  |  | *Columbina talpacoti* | Ruddy Ground Dove | | 5 | 1 | | 6 |
|  |  |  | *Leptotila rufaxilla* | Grey-fronted Dove | | 7 | 9 | | 16 |
|  |  |  | *Leptotila verreauxi* | White-tipped Dove | | 7 | 10 | | 17 |
|  |  |  | *Patagioena cayanensis* | Pale-vented Pigeon | | 1 | 2 | | 3 |
|  |  |  | *Zenaida auriculata* | Eared Dove | | - | 3 | | 3 |
|  | Coraciiformes | Alcedinidae | *Chloroceryle aenea* | American Pygmy Kingfisher | | 1 | 2 | | 3 |
|  |  |  | *Chloroceryle amazona* | Amazon Kingfisher | | 3 | 4 | | 7 |
|  |  |  | *Chloroceryle americana* | Green Kingfisher | | 3 | 10 | | 13 |
|  |  |  | *Megaceryle torquata* | Ringed Kingfisher | | 2 | 1 | | 3 |
|  |  | Cuculidae | *Coccycua minuta* | Little Cuckoo | | 1 | 1 | | 2 |
|  |  |  | *Crotophaga ani* | Smooth-billed Ani | | 25 | - | | 25 |
|  |  |  | *Crotophaga major* | Greater Ani | | 4 | - | | 4 |
|  | Eurypygiformes | Eurypygidae | *Eurypyga helias* | Sunbittern | | - | 3 | | 3 |
|  | Falconiformes | Falconidae | *Daptrius chimachima* | Yellow-headed Caracara | | 1 | 1 | | 2 |
|  | Galliformes | Cracidae | *Ortalis ruficauda* | Rufous-vented Chachalaca | | 1 | - | | 1 |
|  |  | Numididae | *Numida meleagris* | Helmeted Guineafowl | | 7 | 4 | | 11 |
|  |  | Odontophoridae | *Colinus cristatus* | Crested Bobwhite | | 1 | - | | 1 |
|  |  | Phasianidae | *Gallus gallus* | Red Junglefowl | | 45 | 37 | | 82 |
|  |  |  | *Meleagris gallopavo* | Wild Turkey | | 3 | - | | 3 |
|  |  |  | *Pavo cristatus* | Indian Peafowl | | 1 | - | | 1 |
|  | Gruiformes | Rallidae | *Aramides cajanea* | Grey-necked Wood Rail | | 1 | 11 | | 12 |
|  |  |  | *Porphyrio martinica* | Purple Gallinule | | 1 | - | | 1 |
|  |  |  | *Porphyrio flavirostris* | Azure Gallinule | | 2 | - | | 2 |
|  | Nyctibiiformes | Nyctibiidae | *Nyctibius grandis* | Great Potoo | | 3 | - | | 3 |
|  | Opisthocomiformes | Opisthocomidae | *Opisthocomus hoazin* | Hoatzin | | 4 | - | | 4 |
|  | Passeriformes | Donacobiidae | *Donacobius atricapilla* | Black-capped Donacobius | | 2 | - | | 2 |
|  |  | Fringillidae | *Euphonia xanthogaster* | Orange-bellied Euphonia | | 1 | - | | 1 |
|  |  | Furnariidae | *Certhiaxis cinnamomeus* | Yellow-chinned Spinetail | | 1 | - | | 1 |
|  |  |  | *Cranioleuca vulpina* | Parker's Spinetail | | 1 | - | | 1 |
|  |  |  | *Dendrocincla fuliginosa* | Plain-brown Woodcreeper | | 1 | - | | 1 |
|  |  |  | *Dendroplex picus* | Straight-billed Woodcreeper | | 5 | 1 | | 6 |
|  |  |  | *Phacellodomus rufifrons* | Rufous-fronted Thornbird | | 3 | 4 | | 7 |
|  |  |  | *Synallaxis albescens* | Pale-breasted Spinetail | | - | 1 | | 1 |
|  |  |  | *Xiphorhynchus obsoletus* | Striped Woodcreeper | | - | 1 | | 1 |
|  |  | Hirundinidae | *Pygochelidon cyanoleuca* | Blue-and-white Swallow | | - | 1 | | 1 |
|  |  | Icteridae | *Cacicus cela* | Yellow-rumped Cacique | | 1 | 5 | | 6 |
|  |  |  | *Gymnomystax mexicanus* | Oriole Blackbird | | 2 | 5 | | 7 |
|  |  |  | *Icterus icterus* | Venezuelan Troupial | | 2 | - | | 2 |
|  |  |  | *Icterus nigrogularis* | Yellow Oriole | | 4 | 2 | | 6 |
|  |  |  | *Quiscalus lugubris* | Carib Grackle | | - | 10 | | 10 |
|  |  |  | *Sturnella magna* | Eastern Meadowlark | | - | 1 | | 1 |
|  |  |  | *Sturnella militaris* | Red-breasted Meadowlark | | 1 | - | | 1 |
|  |  | Mimidae | *Mimus gilvus* | Tropical Mockingbird | | 7 | - | | 7 |
|  |  | Parulidae | *Parkesia noveboracensis* | Northern Waterthrush | | 1 | - | | 1 |
|  |  | Passerellidae | *Ammodramus aurifrons* | Yellow-browed Sparrow | | 1 | - | | 1 |
|  |  | Thamnophilidae | *Formicivora grisea* | White-fringed Antwren | | 1 | 1 | | 2 |
|  |  |  | *Sakesphorus canadensis* | Black-crested Antshrike | | 1 | 4 | | 5 |
|  |  |  | *Thamnophilus doliatus* | Barred Antshrike | | - | 4 | | 4 |
|  |  | Thraupidae | *Coereba flaveola* | Bananaquit | | 2 | - | | 2 |
|  |  |  | *Paroaria nigrogenis* | Masked Cardinal | | 13 | 8 | | 21 |
|  |  |  | *Ramphocelus carbo* | Silver-beaked Tanager | | 11 | 7 | | 18 |
|  |  |  | *Saltator olivascens* | Olive-gray Saltator | | 3 | 2 | | 5 |
|  |  |  | *Saltator coerulescens* | Greyish Saltator | | 11 | 3 | | 14 |
|  |  |  | *Saltator maximus* | Buff-throated Saltator | | 2 | - | | 2 |
|  |  |  | *Sicalis columbiana* | Orange-fronted Yellow Finch | | 1 | - | | 1 |
|  |  |  | *Sicalis flaveola* | Saffron Finch | | 4 | 8 | | 12 |
|  |  |  | *Sporophila angolensis* | Chestnut-bellied Seedeater | | 1 | - | | 1 |
|  |  |  | *Sporophila intermedia* | Grey Seedeater | | 1 | 5 | | 6 |
|  |  |  | *Sporophila sp.* | Seed Finch | | - | 1 | | 1 |
|  |  |  | *Stilpnia cayana* | Burnished-buff Tanager | | 2 | 2 | | 4 |
|  |  |  | *Tachyphonus rufus* | White-lined Tanager | | - | 2 | | 2 |
|  |  |  | *Thraupis episcopus* | Blue-grey Tanager | | 10 | 11 | | 21 |
|  |  |  | *Thraupis palmarum* | Palm Tanager | | 23 | 1 | | 24 |
|  |  | Turdidae | *Catharus ustulatus* | Swainson's Thrush | | 1 | - | | 1 |
|  |  |  | *Turdus ignobilis* | Black-billed Thrush | | 5 | 4 | | 9 |
|  |  |  | *Turdus leucomelas* | Pale-breasted Thrush | | - | 5 | | 5 |
|  |  |  | *Turdus nudigenis* | Bare-eyed Thrush | | - | 11 | | 11 |
|  |  | Tyrannidae | *Attila cinnamomeus* | Cinnamon Attila | | 2 | - | | 2 |
|  |  |  | *Elaenia chiriquensis* | Lesser Elaenia | | 1 | 4 | | 5 |
|  |  |  | *Elaenia flavogaster* | Yellow-bellied Elaenia | | 2 | 2 | | 4 |
|  |  |  | *Elaenia parvirostris* | Small-billed Elaenia | | 2 | 5 | | 7 |
|  |  |  | *Fluvicola pica* | Pied Water Tyrant | | 2 | 1 | | 3 |
|  |  |  | *Leptopogon amaurocephalus* | Sepia-capped Flycatcher | | - | 1 | | 1 |
|  |  |  | *Megarynchus pitangua* | Boat-billed Flycatcher | | - | 2 | | 2 |
|  |  |  | *Myiarchus cephalotes* | Pale-edged Flycatcher | | - | 5 | | 5 |
|  |  |  | *Myiarchus ferox* | Short-crested Flycatcher | | 2 | 5 | | 7 |
|  |  |  | *Myiopagis gaimardii* | Forest Elaenia | | - | 1 | | 1 |
|  |  |  | *Myiarchus tyrannulus* | Brown-crested Flycatcher | | 1 | 1 | | 2 |
|  |  |  | *Myiarchus swainsoni* | Swainson's Flycatcher | | 1 | - | | 1 |
|  |  |  | *Myiodynastes maculatus* | Streaked Flycatcher | | 2 | - | | 2 |
|  |  |  | *Myiozetetes cayenensis* | Rusty-margined Flycatcher | | 4 | 21 | | 25 |
|  |  |  | *Phelpsia inornata* | Drab Water Tyrant | | 1 | 7 | | 8 |
|  |  |  | *Pitangus lictor* | Lesser Kiskadee | | 5 | 2 | | 7 |
|  |  |  | *Pitangus sulphuratus* | Great Kiskadee | | 13 | 13 | | 26 |
|  |  |  | *Rhytipterna simplex* | Grayish Mourner | | - | 1 | | 1 |
|  |  |  | *Tyrannus melancholicus* | Tropical Kingbird | | 24 | 13 | | 37 |
|  |  |  | *Tyrannus savana* | Fork-tailed Flycatcher | | 31 | 2 | | 33 |
|  |  | Vireonidae | *Hylophilus flavipes* | Scrub Greenlet | | - | 2 | | 2 |
|  |  |  | *Cyclarhis gujanensis* | Rufous-browed Peppershrike | | - | 1 | | 1 |
|  | Pelecaniformes | Ardeidae | *Ardea cocoi* | Cocoi Heron | | 1 | - | | 1 |
|  |  |  | *Bubulcus ibis* | Cattle Egret | | 2 | 9 | | 11 |
|  |  |  | *Butorides striata* | Striated Heron | | 2 | - | | 2 |
|  |  |  | *Tigrisoma lineatum* | Rufescent Tiger-Heron | | 1 | - | | 1 |
|  |  |  | *Egretta caerulea* | Little Blue Heron | | 2 | 4 | | 6 |
|  |  |  | *Egretta thula* | Snowy Egret | | - | 2 | | 2 |
|  |  | Threskiornithidae | *Eudocimus ruber* | Scarlet Ibis | | - | 6 | | 6 |
|  |  |  | *Phimosus infuscatus* | Bare-faced Ibis | | 1 | 2 | | 3 |
|  |  |  | *Platalea ajaja* | Roseate Spoonbill | | - | 17 | | 17 |
|  |  |  | *Theristicus caudatus* | Buff-necked Ibis | | - | 1 | | 1 |
|  | Piciformes | Galbulidae | *Brachygalba goeringi* | Pale-headed Jacamar | | - | 1 | | 1 |
|  |  |  | *Galbula ruficauda* | Rufous-tailed Jacamar | | 5 | - | | 5 |
|  |  | Picidae | *Colaptes punctigula* | Spot-breasted Woodpecker | | 4 | 1 | | 5 |
|  |  |  | *Melanerpes rubricapillus* | Red-crowned Woodpecker | | 2 | - | | 2 |
|  |  |  | *Picumnus squamulatus* | Scaled Piculet | | 1 | 1 | | 2 |
|  |  |  | *Veniliornis fumigatus* | Smoky-brown Woodpecker | | - | 1 | | 1 |
|  | Psittaciformes | Psittacidae | *Ara macao* | Scarlet Macaw | | 1 | - | | 1 |
|  |  |  | *Forpus conspicillatus* | Spectacled Parrotlet | | 5 | - | | 5 |
|  | Strigiformes | Strigidae | *Athene cunicularia* | Burrowing Owl | | 9 | 10 | | 19 |
|  |  |  | *Megascops choliba* | Tropical Screech Owl | | 1 | 1 | | 2 |
|  | Suliformes | Anhingidae | *Anhinga anhinga* | Anhinga | | 1 | - | | 1 |
|  |  | Phalacrocoracidae | *Phalacrocorax brasilianus* | Neotropic Cormorant | | - | 2 | | 2 |
| **Total group** | | | | | | **588** | | **628** | **1216** |
| **Total** | | | | | | | | | **2553** |
